# Exposure to ionizing radiation induced persistent gene expression changes in mouse mammary gland

**DOI:** 10.1186/1748-717X-7-205

**Published:** 2012-12-05

**Authors:** Kamal Datta, Daniel R Hyduke, Shubhankar Suman, Bo-Hyun Moon, Michael D Johnson, Albert J Fornace

**Affiliations:** 1 Department of Biochemistry and Molecular & Cellular Biology, Georgetown University, 3970 Reservoir Rd, Washington, DC, NW 20057-1468, USA; 2 Lombardi Comprehensives Cancer Center, Georgetown University, 3970 Reservoir RD, Washington, DC, NW 20057, USA; 3 Center of Excellence In Genomic Medicine Research (CEGMR), King Abdulaziz University, Jeddah, Saudi Arabia

**Keywords:** Radiation exposure, Mouse mammary gland, Gene expression, Persistent microarray changes.

## Abstract

**Background:**

Breast tissue is among the most sensitive tissues to the carcinogenic actions of ionizing radiation and epidemiological studies have linked radiation exposure to breast cancer. Currently, molecular understanding of radiation carcinogenesis in mammary gland is hindered due to the scarcity of *in vivo* long-term follow up data. We undertook this study to delineate radiation-induced persistent alterations in gene expression in mouse mammary glands 2-month after radiation exposure.

**Methods:**

Six to eight week old female C57BL/6J mice were exposed to 2 Gy of whole body γ radiation and mammary glands were surgically removed 2-month after radiation. RNA was isolated and microarray hybridization performed for gene expression analysis. Ingenuity Pathway Analysis (IPA) was used for biological interpretation of microarray data. Real time quantitative PCR was performed on selected genes to confirm the microarray data.

**Results:**

Compared to untreated controls, the mRNA levels of a total of 737 genes were significantly (p<0.05) perturbed above 2-fold of control. More genes (493 genes; 67%) were upregulated than the number of downregulated genes (244 genes; 33%). Functional analysis of the upregulated genes mapped to cell proliferation and cancer related canonical pathways such as ‘ERK/MAPK signaling’, ‘CDK5 signaling’, and ‘14-3-3-mediated signaling’. We also observed upregulation of breast cancer related canonical pathways such as ‘breast cancer regulation by Stathmin1’, and ‘HER-2 signaling in breast cancer’ in IPA. Interestingly, the downregulated genes mapped to fewer canonical pathways involved in cell proliferation. We also observed that a number of genes with tumor suppressor function (GPRC5A, ELF1, NAB2, Sema4D, ACPP, MAP2, RUNX1) persistently remained downregulated in response to radiation exposure. Results from qRT-PCR on five selected differentially expressed genes confirmed microarray data. The PCR data on PPP4c, ELF1, MAPK12, PLCG1, and E2F6 showed similar trend in up and downregulation as has been observed with the microarray.

**Conclusions:**

Exposure to a clinically relevant radiation dose led to long-term activation of mammary gland genes involved in proliferative and metabolic pathways, which are known to have roles in carcinogenesis. When considered along with downregulation of a number of tumor suppressor genes, our study has implications for breast cancer initiation and progression after therapeutic radiation exposure.

## Background

We know from epidemiological studies that exposure to ionizing radiation (IR) is one of the major risk factors for breast cancer especially if exposure occurs at a young age [1,2]. Exposure to radiation, which is increasingly used in diagnostic and therapeutic interventions, is unavoidable and is predicted to pose carcinogenic risk [3,4]. Radiation induces a myriad of damage to biomolecules including DNA and the carcinogenic potential of radiation has been attributed to alterations in the expression of genes, which could induce proliferation and provide survival advantage to cells. Changes in gene expression in response to radiation have been reported to vary depending on the dose and the tissue exposed; and, there are significant differences in acute and delayed responses to IR exposure [5]. Delayed effects of IR on gene expression are not only associated with cancer initiation and promotion in normal cells but are also known to play a critical role in the development of resistance to therapy in cancer cells [5]. Therefore, radiation treatment of breast cancer has on the one hand the potential for cancer recurrence and on the other the risk for developing new cancer in the contralateral breast [4]. Despite the fact that radiation exposure causes persistent changes in gene expression, data on radiation-induced long-term alterations in gene expression in mammary tissues are of limited availability. Furthermore, there are no reports in the literature of long-term microarray-based studies of changes in mouse mammary gland gene expression after exposure to radiation doses relevant to therapeutic procedures. Considering the carcinogenic potential of radiation exposure to mammary glands, it is important to understand how radiation modulates long-term gene expression changes and how these changes relate to oncogenic signaling pathways known to be involved in breast cancer initiation and progression. Therefore, the goals of this study were to characterize persistent transcriptomic alterations and explore how these alterations relate to biological functions in whole body exposed 6 to 8 week old female C57BL6/J mouse mammary glands 2 months after exposure to 2 Gy γ radiation.

## Methods

### Mice and irradiation

Mice (C57BL/6J, female, 6-8 week old) were purchased from Jackson Laboratories (Bar Harbor, ME, USA) and were housed at the Georgetown University’s (GU) animal facility. All the animal procedures were performed as per protocol approved by GU Animal Care and Use Committee (GUACUC). Mice were placed in a circular pie shaped, well-ventilated plastic mouse holder, the mouse holder with mice was positioned on a rotating turntable inside the irradiator, and mice were exposed to γ radiation using a ^137^Cs source. Radiation dose was delivered at a rate of 1 Gy/min and a total dose of 2 Gy was delivered to the whole body and the control groups were sham irradiated. After irradiation mice were returned to their home cage, and monitored regularly.

### RNA isolation

Mice were euthanized by asphyxiation using CO_2_ as per GUACUC approved protocol and mammary glands were surgically removed 2 month after radiation exposure for RNA extraction. For RNA extraction, we used no. 4 mammary gland from the left side of each mouse and four such mice were used in each experimental group. Total RNA was isolated with Trizol reagent (Invitrogen, Carlsbad, CA) from fresh or flash frozen mammary glands followed by purification over an RNeasy column according to the manufacturer’s instructions (Qiagen, Germantown, MD) and stored at -80°C for further use. RNA quality was determined using a Bioanalyzer (Agilent Technologies, Palo Alto, CA).

### Microarray analysis

Cy3-labeled cRNA was generated from 300 ng total RNA using Agilent’s Low RNA Input Linear Amplification PLUS kit. Cy3-labeled cRNA (1.65 μg) was hybridized to Agilent’s Mouse Whole Genome arrays (G4122F) with Agilent’s GEx Hybridization HI-RPM Buffer. The samples were hybridized for 16 hours in a rotisserie hybridization oven (G2545A, Agilent Technologies, Santa Clara, CA, USA), then washed with Agilent’s gene expression wash buffers and scanned with an Agilent DNA Microarray Scanner 2505A in an ozone hood. Subsequent image analysis, data extraction, and background corrections were performed with Agilent’s Feature Extraction Software 9.1. Extracted data were then analyzed using GeneSpring GX 10.0 (Agilent Technologies) to identify genes that had significantly changed expression between control and the irradiated group. Although small sample size (3 to 5 biological replicates) conventionally used in microarray experiments has its limitations, the large data sets used in comparative analysis provide enough statistical power to derive biologically meaningful inferences from the results (discussed in Prolla, 2002 [6]). Genes considered significantly (p<0.05, t-test with Benjamini-Hochberg correction [7]) and differentially (fold change ≥2.0) regulated were listed separately.

### Pathway analysis for biological functions

Significantly perturbed gene lists generated from microarray analysis were uploaded onto Ingenuity Pathway Analysis (IPA, Ingenuity Systems Inc., Redwood City, CA, USA) for assignment of biological function as well as for identifying perturbed signal transduction networks. IPA combines the uploaded data and the Ingenuity Pathways Knowledge Base (IPKB), created with information from available literature, to identify biological networks that are significantly over-represented in the gene expression data. Right-tailed Fisher's exact test is applied to determine the level of significance for each network and the p-value is displayed as score, which is the negative log of that p-value. A score of 8 indicates that there is a 1 in 10^8^ chance that the focus genes are together in a network due to random chance alone. Furthermore, IPA uses a built-in library to identify association between the uploaded genes with the canonical pathways present in the knowledge base. In addition to providing a significance score, IPA also calculates the number of genes within a dataset that are present in each significantly perturbed pathway.

### Quantitative real time PCR (qRT-PCR)

For confirmation of microarray data by qRT-PCR, cDNA was generated from 1.5 μg RNA using the RT^2^ First Strand Kit as per recommendation (SA Biosciences, Frederick, MD, USA). Primers for PPP4c (protein phosphatase 4, catalytic subunit; Cat# PPM28940A), MAPK12 (mitogen-activated protein kinase 12; Cat#PPM04541C), PLCG1 (phospholipase C, gamma 1; Cat#PPM004022B), and β-actin (Cat# PPM02945A) were obtained from SA Biosciences. Primers for E2F6 (Forward primer: 5’-GATGGCATCGAACTGGTGGAA-3’; reverse primer: 5’-CCCCAAAGTTGTTCAGGTCAG-3’ [8]), and ELF1 (Forward primer: 5’-TGTCCAACAGAACGACCTAGT-3’; reverse primer: 5’ CACACAAGCTAGACCAGCATAA-3’ [8]) were obtained from Eurofins MWG Operon (Huntsville, AL, USA). Primer for NFkβ, which is the nodal molecule of the top scoring molecular pathway network identified from the microarray data set by IPA, was also obtained from Eurofins MWG Operon (Forward primer: 5’-AGCACATAGATGAACTCCG-3’; reverse primer: 5’- CTGTAAAGCTGAGTTTGCG-3’ [9]). Using the RT^2^ SYBR Green/Fluorescein qPCR Master mix (SA Biosciences) qRT-PCR was performed on an ABI 7900HT (Applied Biosystems, Carlsbad, CA, USA) platform with temperature settings: 95°C (denaturation) for 10 min and then 40 cycles of 95°C for 15 sec, annealing/extension at 60°C for 1 min. The fold change was calculated using β-actin as an endogenous control following the comparative *Ct* (ΔΔ*Ct*) method as described previously [10]. Results were expressed relative to control samples, and three biological replicates were used in each experimental group. The error bar represents standard error of mean (SEM).

## Results

### Greater number of genes showed persistent upregulation following radiation exposure

**Figure 1:**
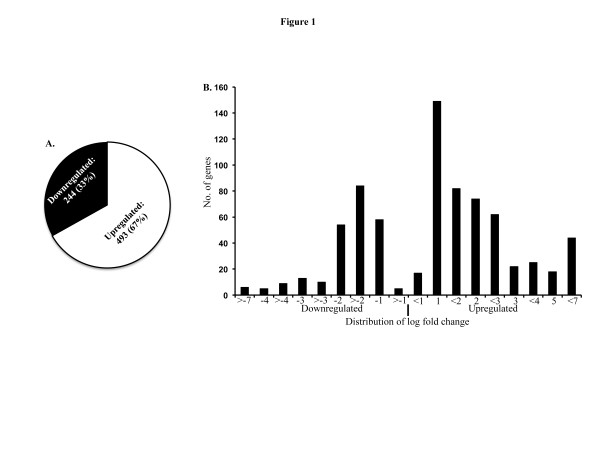
**Exposure to ionizing radiation results in persistent perturbations of mammary gland gene expression.A**) Total number of transcripts perturbed 2-month after exposure to 2 Gy of whole body γ radiation. About 67% of the transcriptomes were upregulated and 33% remained downregulated. **B**) Transcripts significantly modulated relative to control (p<0.05 and more than 2-fold change) were plotted showing fold change distribution. Most of the upregulated genes were between 1 and 3 fold and the downregulated genes were clustered mostly between -1 and -2 fold.

Global analysis of microarray data indicated that compared to control the mRNA level of a total of 737 genes remained perturbed 2-month after exposure to 2 Gy of γ radiation. While 67% (493 genes) of the genes were upregulated, we observed that only 33% (244 genes) of the genes were downregulated (Figure 1A). When we assessed the scale of variation in the significantly perturbed genes’ list (relative to control p<0.05 and above 2-fold), the majority of the upregulated fold changes were between 1 and 3 fold and the majority of the downregulated fold changes were between 1 and 2 fold (Figure 1B).

### Quantitative real time PCR confirmation of microarray data

**Figure 2:**
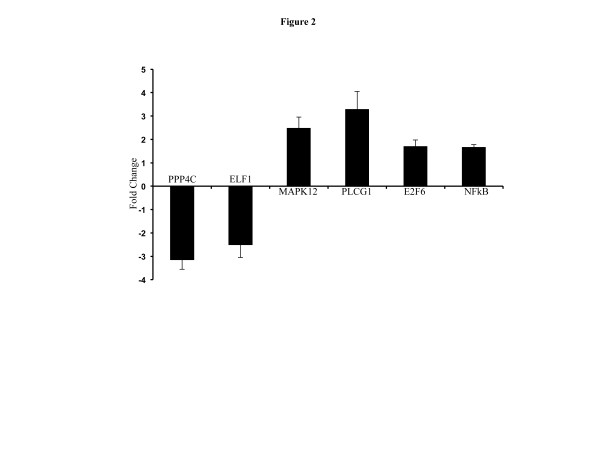
**Confirmation of microarray data by PCR analysis.** Results of PCR analysis of 2 downregulated (PPP4C, and ELF1) and 3 upregulated (MAPK12, PLCG1, and E2F6) genes showed a trend similar to the microarray fold change. NFkβ is the nodal molecule of the top upregulated network.

We performed qRT-PCR on five selected differentially expressed genes to confirm microarray data. The PCR data on PPP4c, ELF1, MAPK12, PLCG1, and E2F6 exhibited a trend similar to our microarray measurements (Figure 2). Compared to control, PPP4c (fold change -3.14 ± 0.41 standard error of mean (SEM); p<0.001; microarray fold change -1.54) and ELF1 (fold change -2.49±0.55; p<0.003; microarray fold change -1.78) was downregulated and were consistent with microarray results. Also, in agreement with our microarray data, we observed upregulation of MAPK12 (2.47±0.47; p<0.02; microarray fold change 1.85), PLCG1 (3.28±0.76; p<0.02; microarray fold change 1.96), and E2F6 (1.69±0.28; p<0.04; microarray fold change 1.7) expression. We also performed qRT-PCR of NFkβ, which is the nodal molecule of the highest scoring molecular network obtained from IPA (fold change 1.65 ± 0.12; p<0.03 in γ irradiated samples compared to control; Figure 2).

### Pathway analysis shows upregulation of metabolic and proliferative pathways

**Figure 3:**
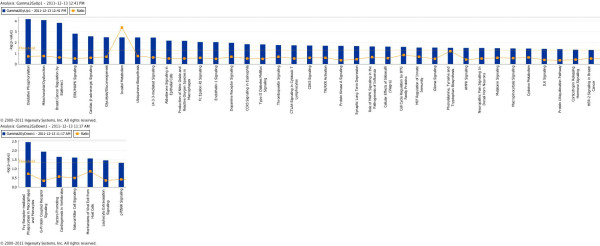
**Radiation-induced alterations in canonical pathways identified by Ingenuity Pathway Analysis.A**) A total of 37 canonical pathways were significantly upregulated (p<0.05 indicated by threshold line). **B**) A total of 7 canonical pathways were downregulated (p<0.05).

To identify which pathways were perturbed 2 months after radiation exposure, we subjected the up and downregulated gene sets to IPA. The results of the analysis associating altered genes to specific canonical pathways are presented in Table 1 (Upregulated) and 2 (Downregulated), and also in Figure 3A (Upregulated) and B (Downregulated). While the upregulated gene sets were significantly associated with 37 canonical pathways (35 presented in Table 1) with a p value cutoff of 0.05 (corresponds to -log p value of 1.3), the downregulated genes were mapped to 7 pathways with similar level of significance (Table 2). Most of the upregulated pathways were involved in cellular growth and proliferation including ‘ERK/MAPK signaling’, ‘14-3-3-mediated signaling’, ‘endothelin-1 Signaling’, ‘protein kinase A signaling’, ‘AMPK signaling’, and ‘corticotropin releasing hormone signaling’. At least two pathways related to breast cancer – ‘breast cancer regulation by Stathmin1’ and ‘HER-2 signaling in breast cancer’ – were also found to be upregulated. Metabolism was, also, a common theme among the upregulated pathways with the highest scoring pathways being ‘oxidative phosphorylation’ and ‘mitochondrial dysfunction’. Additionally, ‘glycolysis/gluconeogenesis’, ‘inositol metabolism’, and ‘ubiquinone biosynthesis’ were in the top 10 upregulated pathways. The downregulated gene sets were mapped to fewer canonical pathways none of which are associated with cellular metabolism or proliferation except for the ‘G-protein coupled receptor signaling’. All the up and downregulated canonical pathways obtained from the IPA are presented in Additional files 1 (Upregulated) and 2 (Downregulated) for reference.

**Table 1:** Significant upregulation of pathways involved in cell proliferation, metabolism and breast cancer

**Ingenuity Canonical Pathways**	**-log(p-value)**	**Molecules**
Oxidative phosphorylation	4.16	ATP6V0E2, NDUFC1, COX6B1, NDUFS8, COX10, COX5A, NDUFS6, NDUFA12, COX7A1, ATP5G3
Mitochondrial dysfunction	4.06	COX6B1, NDUFS8, COX10, CPT1B, COX5A, TRAK1, NDUFS6, NDUFA12, MAPK12, COX7A1
Breast cancer regulation by Stathmin1	3.81	PPP1R14C, PIK3C2B, E2F6, ADCY2, PRKCQ, CAMK2A, TUBA8, PPM1L, PPP2R5B, TUBA4A, PPP1R3A, TUBA3C/TUBA3D
ERK/MAPK signaling	2.81	PLA2G4E, PPP1R14C, PIK3C2B, TLN2, PLA2G12A, HSPB2, PPM1L, PPP2R5B, PLCG1, PPP1R3A
Cardiac β-adrenergic signaling	2.56	PPP1R14C, ADCY2, PPM1L, PPP2R5B, PDE4A, AKAP6, PPP1R3A, PDE4D
Glycolysis/gluconeogenesis	2.47	PGK1, PGM1, ALDOA, TPI1, Gapdh (includes others), LDHB
Inositol metabolism	2.46	ALDOA, TPI1
Ubiquinone biosynthesis	2.46	NDUFC1, NDUFS8, UFSP1, NDUFS6, NDUFA12
14-3-3-mediated signaling	2.44	PIK3C2B, PRKCQ, TUBA8, TUBA4A, PLCG1, TUBA3C/TUBA3D, MAPK12
Aldosterone signaling in epithelial cells	2.16	PIK3C2B, SACS, PRKCQ, HSPA1L, DNAJC27, HSPB2, PLCG1, DNAJB5
Production of nitric oxide and reactive oxygen species in macrophages	2.14	PPP1R14C, PIK3C2B, PRKCQ, PPM1L, PPP2R5B, PLCG1, PPP1R3A, MAPK12
Fc epsilon RI signaling	2.03	PLA2G4E, PIK3C2B, PRKCQ, PLA2G12A, PLCG1, MAPK12
Endothelin-1 signaling	2.03	PLA2G4E, PIK3C2B, ADCY2, PRKCQ, PLA2G12A, GNAO1, PLCG1, MAPK12
Dopamine receptor signaling	1.96	PPP1R14C, ADCY2, PPM1L, PPP2R5B, PPP1R3A
CCR3 signaling in eosinophils	1.83	PLA2G4E, PIK3C2B, PRKCQ, PLA2G12A, CFL2, MAPK12
Type II diabetes mellitus signaling	1.81	PIK3C2B, RKCQ, PRKAB2, MAPK12, ADIPOR1, KCNJ11
Thrombopoietin signaling	1.75	PIK3C2B, THPO, PRKCQ, PLCG1
CTLA4 signaling in cytotoxic T lymphocytes	1.73	PIK3C2B, AP1S2, PPM1L, PPP2R5B, PLCG1
CDK5 signaling	1.71	PPP1R14C, ADCY2, PPM1L, PPP2R5B, PPP1R3A
TR/RXR activation	1.69	PIK3C2B, RXRG, SLC16A3, UCP1, PPARGC1A
Protein kinase A signaling	1.68	PPP1R14C, ADCY2, PRKCQ, CAMK2A, HIST1H1A, PDE4A, PLCG1, AKAP6, PPP1R3A, TTN, PDE4D
Synaptic long term depression	1.65	PLA2G4E, PRKCQ, PLA2G12A, PPM1L, GNAO1, PPP2R5B
Role of MAPK signaling in the pathogenesis of influenza	1.62	PLA2G4E, PLA2G12A, MAPK12, RABGEF1
Cell cycle regulation by BTG family proteins	1.58	E2F6, PPM1L, PPP2R5B
MIF regulation of innate immunity	1.52	PLA2G4E, PLA2G12A, MAPK12
glioma signaling	1.52	PIK3C2B, E2F6, PRKCQ, CAMK2A, PLCG1
Phenylalanine, tyrosine and tryptophan biosynthesis	1.50	GOT1, GOT2
AMPK signaling	1.48	PIK3C2B, PRKAB2, CPT1B, PPM1L, PPP2R5B, MAPK12
Melatonin signaling	1.46	PRKCQ, CAMK2A, GNAO1, PLCG1
Macropinocytosis signaling	1.44	PIK3C2B, PRKCQ, PLCG1, ITGB6
Cysteine metabolism	1.43	GOT1, GOT2, LDHB
ILK signaling	1.40	PIK3C2B, CFL2, PPM1L, PPP2R5B, MAPK12, ITGB6, MYH1
Protein ubiquitination pathway	1.38	SACS, USP15, HSPA1L, USP13, UBE2B, DNAJC27, HSPB2, USP2, DNAJB5
Corticotropin releasing hormone Signaling	1.32	ADCY2, PRKCQ, GNAO1, PLCG1, MAPK12
HER-2 signaling in breast cancer	1.30	PIK3C2B, PRKCQ, PLCG1, ITGB6

**Table 2:** Few pathways were downregulated and fewer were involved in cellular proliferation

**Ingenuity Canonical Pathways**	**-log(p-value)**	**Molecules**
Fcγ receptor-mediated phagocytosis in macrophages and monocytes	2.44	EZR, LYN, ACTG1, PRKCB
G-Protein coupled receptor signaling	1.92	PRLHR, GPR173, PTGIR, GPRC5A, GLP2R, FZD3, GPR113, ADCY7, CELSR2, PRKCB
Factors promoting cardiogenesis in vertebrates	1.64	FZD3, TDGF1, PRKCB
Natural killer cell signaling	1.60	SH3BP2, Klra4 (includes others), PRKCB
Mechanisms of viral exit from host cells	1.55	ACTG1, PRKCB
Leukocyte extravasation signaling	1.45	EZR, MMP11, ACTG1, PRKCB
p70S6K signaling	1.32	LYN, RPS6, PRKCB

**Figure 4:**
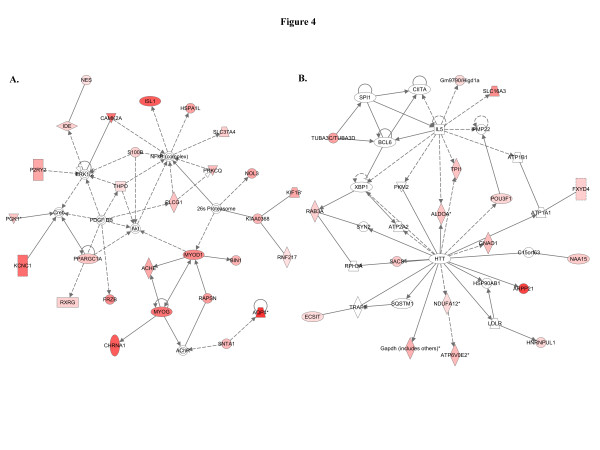
**IPA identified two top signaling networks with genes involved in carcinogenesis.A**) The first network is cellular growth and development network with NFkB as the nodal molecule. At least 12 (ACHE, HSPA1L, MYOD1, NES, NOL3, P2RY2, PGK1, PLCG1, PPARGC1A, PRKCQ, S100B, and SNTA1) genes from this upregulated network are associated with initiation and progression of human cancer. **B**) The second network is genetic disorder, neurological disease, skeletal and muscular disorders and at least 4 upregulated genes (ALDOA, GNAO1, SLC16A3, and TPI1) are known to be involved in cancer.

**Figure 5:**
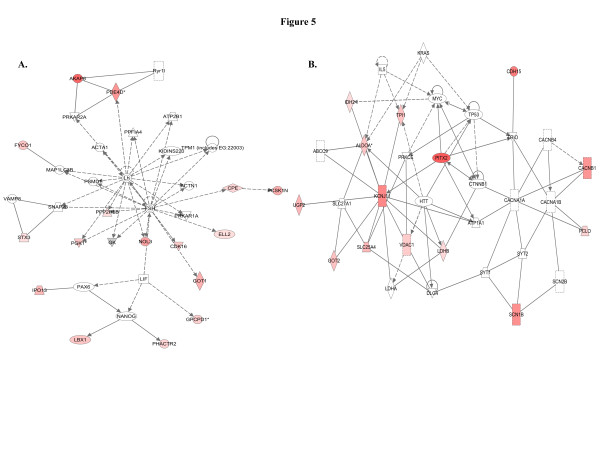
**Upregulation of cellular proliferation and metabolic pathways involved in neoplasia.A**) The third upregulated network involves reproductive system development and function, cellular development, cellular growth and proliferation and upregulation of at least one gene (LBX1) has been associated with cellular transformation. **B**) The fourth upregulated network involves molecular transport, genetic disorder, carbohydrate metabolism and at least 5 (ALDOA, LDHB, SLC16A3, TPI1, VDAC1) genes are related to carcinogenesis.

**Figure 6:**
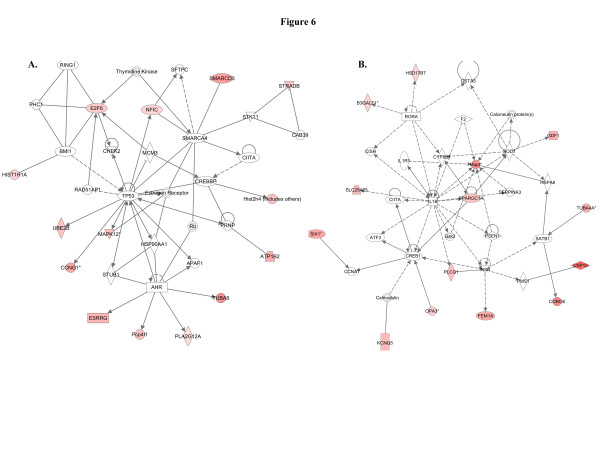
**Cell cycle and cell death related pathways were upregulated. A**) The fifth upregulated network is involved in cell cycle, cell death, hematological system development as well as estrogen signaling such as CCNG1, E2F6, and ESRRG. **B**) The molecules in the sixth network are know to regulate cell death, neurological disease, and tissue morphology.

**Figure 7:**
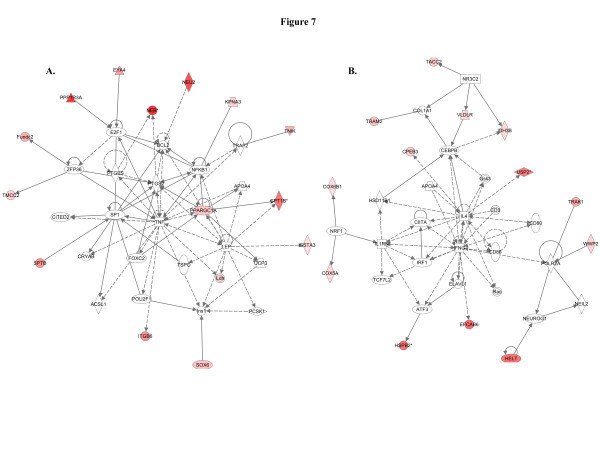
**Two networks were associated with energy production and cell proliferation.A**) Major functions associated with this network are energy production, lipid metabolism, and small molecule biochemistry, and has a score of 16. **B**) The eighth network had a score of 14 and major functions associated with this network are cellular growth and proliferation, hematological system development and function, and tissue development.

**Figure 8:**
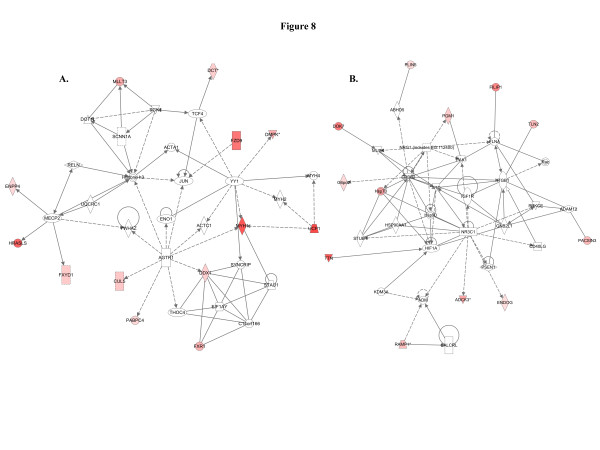
**Cell-to-cell signaling and molecular transport related to carcinogenesis were upregulated.A**) With a score of 14 this pathway network is associated with cellular function and maintenance, molecular transport, cellular assembly and organization. **B**) Eleven genes from the microarray data set were associated with this pathway and identified cell death, cell-to-cell signaling and interaction, nervous system development and function as major functions.

**Figure 9:**
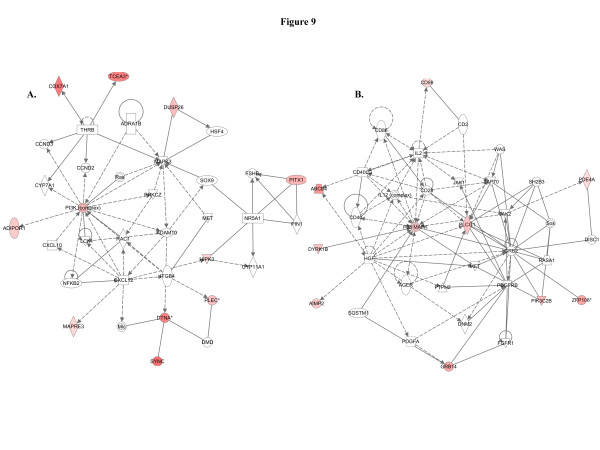
**Cancer and cell death related pathways were upregulated.A**) Genes in this pathway are involved in cancer, tissue development, and embryonic development. **B**) The twelfth network had a score of 8 and 9 genes from the data set are associated with the network which is involved in cellular movement, cell death, and immune cell trafficking.

**Figure 10:**
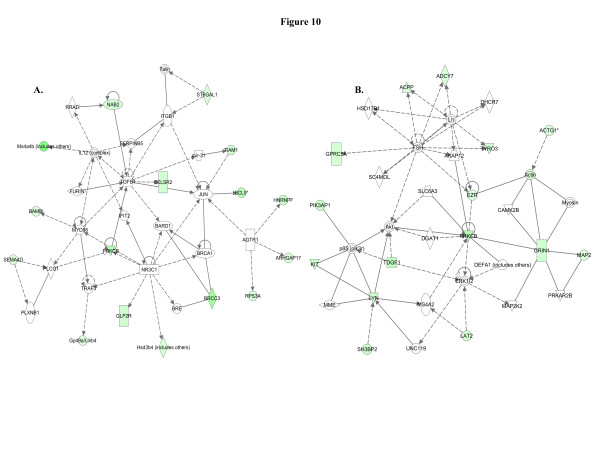
**The downregulated networks may accelerate cellular transformation.A**) The top downregulated network has TGFβ as the nodal molecule and downregulation of at least 8 genes (ARHGAP17, BAMBI, BCL9, BRCC3, CELSR2, NAB2, PRKCB, SEMA4D) in the network facilitates carcinogenesis. **B**) The second downregulated network has 6 genes (ACPP, GPRC5A, KIT, LYN, MAP2, and TDGF1) which may paly a role in cellular transformation.

**Figure 11:**
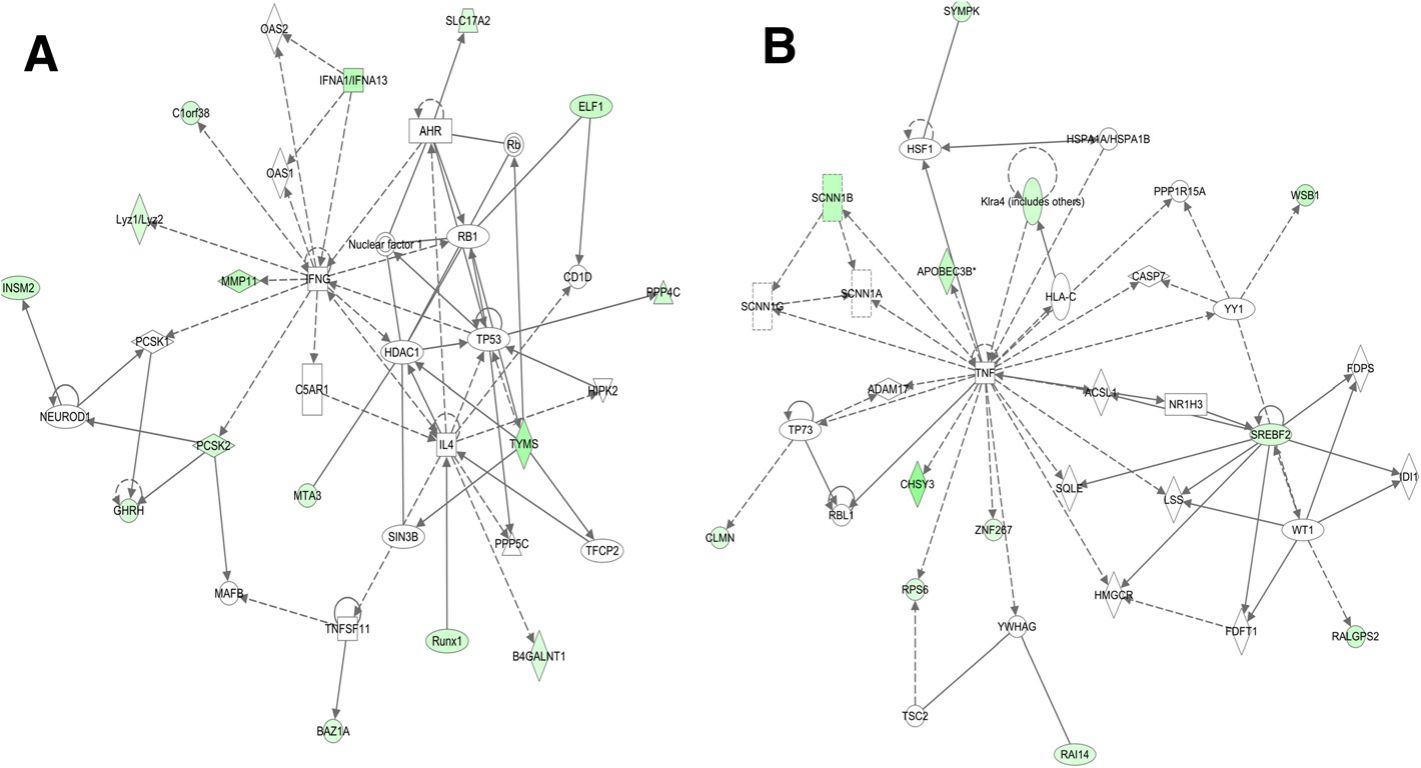
**Balancing promotion and inhibition of cell proliferation in the downregulated networks.A**) Although 1 gene (GHRH) involved in cell proliferation is downregulated in the third network, we observed downregulation of at least 4 key genes (ELF1, MTA3, RUNX1, and TYMS) related to tumor suppressor function. **B**) The fourth downregulated network with a significant score of 18 has TNF as the nodal molecule and downregulation of at least 2 genes (APOBEC3B, CHSY3) are known to promote carcinogenesis.

When we used IPA to map the sets of perturbed genes to gene interaction networks in the Ingenuity Pathways Knowledge Base (IPKB), the upregulated genes were mapped to a total of 23 networks, whereas the downregulated genes were associated with 13 signaling networks. Networks with score ≥8 for the up and downregulated genes are presented in Tables 3 (12 upregulated networks had a score of ≥8) and 4 (4 downregulated networks had a score of ≥8), respectively, and genes from the microarray dataset are identified in bold. Graphical presentation of the up and downregulated networks with a score of ≥8 demonstrates long-term signaling pathway alterations after radiation exposure (Figures 4, 5, 6, 7, 8, 9, 10 and 11). The functions related to upregulated networks ranges from cell death, cell cycle, cell growth and development to carbohydrate and lipid metabolism. Among the upregulated genes, the network with highest score has NFkβ as the nodal molecule (Figure 4A) and, as shown above, qRT-PCR analysis illustrated a 1.65-fold increase in expression of NFkβ in irradiated samples relative to control samples (Figure 2). The network with second highest score has the IL5 (interleukin 5) and HTT (huntingtin) as the nodal molecules and are presented in Figure 4B. The third highest scoring network is involved in the regulation of reproductive system development and function with LH (luteinizing hormone) and FSH (follicle stimulating hormone) as the nodal molecule (Figure 5A) and the fourth is involved in cellular metabolism with KCNJ11 (potassium inwardly-rectifying channel, subfamily J, member 11) as the nodal molecule (Figure 5B). The fifth pathway is involved in cell cycle and cell death and has TP53 as the nodal molecule (Figure 6A). Importantly, the sixth, seventh, and the eighth pathway networks have IL1β (Figure 6B), tumor necrosis factor (TNF; Figure 7A), and IL4/IFNγ (Figure 7B) respectively as the nodal molecules. We also observed that angiotensin receptor type 1 (AGTR1; Figure 8A) and ERBB2 (Figure 8B) are the nodal molecules in ninth and tenth IPA network and has relevance to breast cancer. The eleventh network with PI3K complex (Figure 9A) as its nodal molecule and the twelfth pathway with p38 MAPK (Figure 9B) as its nodal molecule are also known to play important roles in breast cancer initiation and progression. Among the downregulated networks 4 had scores ≥8 and the networks with score in descending order have TGFβ (Figure 10A), PRKCβ (Figure 10B), IFNγ (Figure 11A), and TNF (Figure 11B) as the nodal molecules respectively and are involved in a variety of cellular functions from cell growth and proliferation to lipid metabolism and small molecule biochemistry. Genes, which were significantly associated with the pathway networks in IPA were searched in the literature for their roles in breast cancer and are compiled along with relevant references in Table 5 for upregulated genes and in Table 6 for downregulated genes. A total of 27 upregulated (ACHE [11], HSPA1L [12], NES [13], P2RY2 [14], PLCG1 [15], PPARGC1A [16], PRKCQ [17], SNTA1 [18], ALDOA [19], GNAO1 [20], SLC16A3 [21], TPI1 [22], ELL2 [23], CCNG1 [24], E2F6 [25], ESRRG [26], MAPK12 [27], HSD17B7 [28], TACC2 [29], DDX1 [30], MYH1 [31], UCP1 [32], PACSIN3 [33], PGM1 [34], TLN2 [35], ADIPOR1 [36], PITX1 [37]) and 8 downregulated genes (CELSR2 [38], PRKCB [39], KIT [40], MAP2 [41], TDGF1 [42], ELF1 [43], RUNX1 [44], TYMS [45]) showed association with breast cancer. Furthermore, the data files with the complete list of the upregulated and downregulated networks are presented in Additional files 3 and 4, respectively, for further reference.

**Table 3:** Upregulated networks with score ≥8 are presented

**ID**	**Molecules in Network**	**Score**	**Focus Molecules**	**Top Functions**
1	26sProteasome, **ACHE**, AChR, Akt, **AQP4**, **BIN1**, **CAMK2A, CHRNA1**, Creb,ERK1/2, **FRZB, HSPA1L, IDE**, **ISL1, KCNC1, KIAA0368, KIF1B, MYOD1, MYOG, NES**, NFkB (complex),**NOL3, P2RY2**, PDGF BB, **PGK1, PLCG1, PPARGC1A, PRKCQ, RAPSN, RNF217, RXRG, S100B, SLC37A4, SNTA1, THPO**	44	28	Cell Death, Neurological Disease, Cellular Development
2	**ALDOA, ARPP21**, ATP1A1, ATP1B1, ATP2A2, **ATP6V0E2**, BCL6, C15orf63, CIITA, **ECSIT, FXYD4, Gapdh** (includes others), **Gm9790/Higd1a, GNAO1, HNRNPUL1,** HSP90AB1, HTT, IL5, LDLR, **NAA15, NDUFA12**, PKM2, PMP22, **POU3F1, RAB3A**, RPH3A, **SACS, SLC16A3**, SPI1, SQSTM1, SYN2, **TPI1**, TRAF6, **TUBA3C/TUBA3D**, XBP1	21	17	Genetic Disorder, Neurological Disease, Skeletal and Muscular Disorders
3	ACTA1, ACTN1, **AKAP6**, ATP2B1, **CDK16**, **CPE**, **ELL2**, FSH, **FYCO1**, GK, **GOT1**, **GPCPD1, IPO13**, KIDINS220, **LBX1**, Lh, LIF, MAP1LC3B, NANOG, **NOL3**, PAX6, **PCSK1N**, **PDE4D, PGK1, PHACTR2**, PPFIA4, **PPP2R5B**, PRKAR1A, PRKAR2A, PSMD1, Ryr1l, SNAP23, **STX3**, TPM1 (includes EG:22003), VAMP8	19	16	Reproductive System Development and Function, Cellular Development, Cellular Growth and Proliferation
4	ABCC9, **ALDOA**, ATP1A1, **CACNA1A**, CACNA1B, CACNB1, CACNB4, **CDH15**, CTNNB1, DLG4, **GOT2**, HTT, **IDH2**, IL5, **KCNJ11**, KRAS, LDHA, **LDHB**, MYC, **PCLO, PITX2**, PRKCE, **SCN1B**, SCN2B, **SLC25A4**, SLC27A1, SYT1, SYT2, TP53, **TPI1**, TRIO, **UGP2, VDAC1**	16	14	Molecular Transport, Genetic Disorder, Carbohydrate Metabolism
5	AHR, APAF1, **ATP1B2**, BMI1, CAB39, **CCNG1**, CHEK2, CIITA, CREBBP, **E2F6**, **ESRRG**, Estrogen Receptor, **HIST1H1A, Hist2h4** (includes others), HSP90AA1, **MAPK12**, MCM3, **NFIC, Pcp4l1**, PHC1, **PLA2G12A**, PRNP, RAD51AP1, Rb, RING1, SFTPC, SMARCA4, **SMARCD3**, STK11, **STRADB**, STUB1, Thymidine Kinase, TP53, **TUBA8**, **UBE2B**	16	14	Cell Cycle, Cell Death, Hematological System Development and Function
6	ATF3, **B3GALT1**,Calcineurin protein(s), Calmodulin, CCNA1, CIITA, CISH, **CORO6**, CREB1, CYP8B1, F2, **FEM1A**, **GDF1**, Gsk3, GSTA5, **HSD17B7**, HSPA8, IL1B, IL1R1, INSR, **KCNQ5**, **Mapt, OPA3, PLCG1**, PMS1, **PPARGC1A**, PSEN1, RORA, SATB1, SERPINA3, **SIX1, SLC25A25**, SOD1, **TUBA4A, USP13**	16	14	Cell Death, Neurological Disease, Tissue Morphology
7	ACSL1, APOA4, BCL2, CITED2, **CPT1B**, CRYAB, E2F1, **EYA4**, FOXC2, **Fundc2**, **GSTA3**, Ins1, **ITGB6**, **KPNA3**, Ldh, LEP, **NEB, NEU2**, NFKB1, PCSK1, POU2F1, **PPARGC1A, PPP1R3A**, PTGES, PTGS2, **SOX6**, SP1, **SPTB**, **TMCC2**, TNF, **TNIK**, TRAF2, TSPO, UCP3, ZFP36	16	14	Energy Production, Lipid Metabolism, Small Molecule Biochemistry
8	APOA4, ATF3, CD3, CD80, CD86, CEBPB, CIITA, COL1A1, **COX5A, COX6B1, CPEB3, EFCAB6**, ELAVL1, Gsk3, **HELT**, HSD11B1, **HSPB2, IDH3B**, IFNG, IL4, IL1RN, IRF1, NEIL2, NEUROG1, NR3C2, NRF1, POLR2A, Rac, **TACC2**, TCF7L2, **TRAK1**, **TRAM2, USP2, VLDLR, WWP2**	14	13	Cellular Growth and Proliferation, Hematological System Development and Function, Tissue Development
9	ACTA1, ACTC1, AGTR1, C14orf166, **CUL5, DCT, DDX1, DMPK,** DOT1L, EIF1AY, ENO1, **ENPP4, FXR1, FXYD1, FZD9**, Histone h3, **HRASLS**, JUN, MECP2, **MLLT3, MYH1**, MYH2, MYH4, **PABPC4**, RELN, SCNN1A, SGK1, STAU1, SYNCRIP, TCF4, THOC4, **UCP1**, UQCRC1, YWHAZ, YY1	14	13	Cellular Function and Maintenance, Molecular Transport, Cellular Assembly and Organization
10	ABHD5, ADAM12, **ADCK3**, ADM, CALCRL, CD40LG, **DOK7, ENDOG**, ERBB2, **FILIP1**, FLNA, **G6pd2**, GNB2L1, HIF1A, Hsp70, Hsp90, HSP90AA1, IGF1R, ITGB1, KDM3A, MUSK, NR3C1, NRG1 (includes EG:112400), **PACSIN3**, PAK1, **PGM1**, **PLIN5**, PRKCE, PSEN1, Rac, **RAMP1**, SRC, STUB1, **TLN2, TTN**	11	11	Cell Death, Cell-To-Cell Signaling and Interaction, Nervous System Development and Function
11	ADAM10, **ADIPOR1**, ADRA1B, CCND2, CCND3, **COX7A1**, CXCL10, CXCL12, CYP11A1, CYP7A1, DMD, **DTNA**, **DUSP26**, FSHB, **HIPK3**, HSF4, ITGB4, LCK, MAPK3, **MAPRE3**, MET, Mlc, NFKB2, NR5A1, PI3K (complex), PIN1, **PITX1, PLEC**, PRKCZ, RAC1, Ras, SOX9, **SYNC, TCEA3**, THRB	10	10	Cancer, Tissue Development, Embryonic Development
12	**ABCB4**, AGER, **AIMP2**, CD2, CD28, CD40, **CD59**, CD86, CD40LG, DISC1, DNM2, **DYRK1B**, FGFR1, GRB2, **GRB14**, HGF, IL2, IL12 (complex), JAK1, JAK2, MET, P38 MAPK, **PDE4A**, PDGFA, PDGFRB, **PIK3C2B**, **PLCG1**, PTPN2, ASA1, SH2B3, Sos, SQSTM1, WAS, ZAP70, **ZFP106**	8	9	Cellular Movement, Cell Death, Immune Cell Trafficking

Highlighted genes are from the microarray dataset.

**Table 4:** Downregulated networks with score ≥8 are presented

**ID**	**Molecules in Network**	**Score**	**Focus Molecules**	**Top Functions**
1	AGTR1, **ARHGAP17, BAMBI,** BARD1, **BCL9**, BRCA1, **BRCC3**, BRE, **CELSR2**, FURIN, **GLP2R**, **Gp49a/Lilrb4**, **HNRNPF**, **Hsd3b4** (includes others), IFIT2, IL12 (complex), ITGB1, JUN, mir-31, **Ms4a4b** (includes others), MYD88, **NAB2**, NR3C1, PLCG1, PLXNB1, **PRKCB**, **RPS3A**, RRAD, **SEMA4D**, SERPINB5, **ST6GAL1**, Talin, TGFB1, **TIAM1**, TRAF3	26	16	Cell Morphology, Cellular Growth and Proliferation, Hematological System Development and Function
2	**ACPP, ACTG1**, Actin, **ADCY7**, AKAP12, Akt, CAMK2B, DEFA1 (includes others), DGAT1, DHCR7, ERK1/2, **EZR**, FSH, **GPRC5A**, **GRIN1**, HSD17B1, **KIT, LAT2**, Lh, **LYN, MAP2**, MAP2K2, MME, MS4A2, Myosin, p85 (pik3r), **PIK3AP1**, PRKAR2B, **PRKCB**, SC4MOL, **SH3BP2**, SLC6A3, **TDGF1**, **TYRO3**, UNC119	24	15	Cellular Compromise, Inflammatory Response, Cell Signaling
3	AHR, **B4GALNT1, BAZ1A, C1orf38**, C5AR1, CD1D, **ELF1, GHRH**, HDAC1, HIPK2, **IFNA1/IFNA13**, IFNG,IL4, **INSM2**, **Lyz1/Lyz2**, MAFB, **MMP11**, **MTA3**, NEUROD1, Nuclear factor 1, OAS1, OAS2, PCSK1, **PCSK2, PPP4C**, PPP5C, RB1, Rb**, Runx1**, SIN3B, **SLC17A2**, TFCP2, TNFSF11, TP53, **TYMS**	24	15	Tissue Morphology, Cancer, Gene Expression
4	ACSL1, ADAM17, **APOBEC3B**, CASP7, **CHSY3**, **CLMN**, FDFT1, FDPS, HLA-C, HMGCR, HSF1, HSPA1A/HSPA1B, IDI1, **Klra4** (includes others), LSS, NR1H3, PPP1R15A, **RAI14**, **RALGPS2**, RBL1, **RPS6**, SCNN1A, **SCNN1B**, SCNN1G, SQLE, **SREBF2**, **SYMPK**, TNF, TP73, TSC2, **WSB1**, WT1, YWHAG, YY1, **ZNF267**	18	12	Lipid Metabolism, Small Molecule Biochemistry, Vitamin and Mineral Metabolism

Highlighted genes are from the microarray dataset.

**Table 5:** Genes from upregulated networks: A number of genes from the data set are associated with breast cancer

**Gene**	**Full name**	**Role in breast cancer quoted from literature**
**ACHE**	Acetylcholinesterase	“Tumor size was significantly higher when the ACHE gene was amplified in breast cancer.” [11].
**HSPA1L**	heat shock 70kDa protein 1-like	“….HSPA1L and HSPA2 could represent potential biomarkers to follow up the effectiveness of 17AAG in breast cancer” [12].
**NES**	Nestin	“Among the breast cancer subtypes, nestin is highly expressed in basal breast cancer subtype” [13].
**P2RY2**	purinergic receptor P2Y, G-protein coupled, 2	“P2Y2 receptor-mediated modulation of estrogen-induced proliferation of breast cancer cells” [14].
**PLCG1**	phospholipase C, gamma 1	“Phospholipase Cgamma1 is required for metastasis development and progression” [15].
**PPARGC1A**	peroxisome proliferator-activated receptor gamma, coactivator 1 alpha	“Associations of genetic variants in the estrogen receptor coactivators PPARGC1A, PPARGC1B and EP300 with familial breast cancer” [16].
**PRKCQ**	protein kinase C, theta	“PKCtheta promotes c-Rel-driven mammary tumorigenesis in mice” [17].
**SNTA1**	syntrophin, alpha 1	“….significant increase in expression of SNTA1 protein compared with the normal tissue was observed in breast carcinoma samples” [18].
**ALDOA**	Aldolase A	“…hypoxia-responsive; prognostic significance in breast cancer” [19].
**GNAO1**	guanine nucleotide binding protein (G protein), alpha activating activity polypeptide O	“GNAO1 (Gαo) gene was identified in breast carcinomas and shown to promote oncogenic transformation when introduced into cells” [20].
**SLC16A3**	solute carrier family 16, member 3	“Expression of SLC16A3 gene is higher in breast cancer distant metastasis….” [21].
**TPI1**	triosephosphate isomerase 1	“Glycolytic cancer associated fibroblasts promote breast cancer tumor growth, without a measurable increase in angiogenesis: evidence for stromal-epithelial metabolic coupling” [22].
**ELL2**	elongation factor, RNA polymerase II, 2	ELL2 is a Breast Cancer Antioestrogen Resistance (BCAR) 1 gene which control anti-oestrogen-resistant cell growth resistance (BCAR) [23].
**CCNG1**	cyclin G1	“We have identified cyclin G as being overexpressed in breast and prostate cancer cells” [24].
**E2F6**	E2F transcription factor 6	“E2F6 represses transcription of the *brca1, ctip, art27, hp1*α, and the *rbap48* genes….” [25].
**ESRRG**	Estrogen-related receptor γ	“Estrogen-related receptor γ modulates cell proliferation and estrogen signaling in breast cancer & ERRγ mRNA was up-regulated dose-dependently by estrogen….” [26].
**MAPK12**	mitogen-activated protein kinase 12. Also known as ERK3; ERK6; SAPK3; PRKM12; SAPK-3; P38GAMMA	“…elevated expression of p38γ is associated with lower overall survival of patients with breast cancer” [27].
**HSD17B7**	hydroxysteroid (17-beta) dehydrogenase 7	The stimulation of HSD17B7 expression by estradiol provides a powerful feed-forward mechanism for estradiol biosynthesis in breast cancer cells [28].
**TACC2**	transforming, acidic coiled-coil containing protein 2	“This suggests that increased TACC2 may mediate an oncogenic effect on breast cancer cells and indicates that TACC2 may be a potential therapeutic target” [29].
**DDX1**	DEAD (Asp-Glu-Ala-Asp) box polypeptide 1	“Here, we identify DDX1 RNA overexpression as an independent prognostic marker for early recurrence in primary breast cancer……” [30].
**MYH1**	myosin, heavy chain 1, skeletal muscle, adult	Recently, MYH1 encoding skeletal muscle myosin heavy polypeptide 1 and MYH9 encoding non-muscle myosin heavy chain type A, were identified as candidate breast cancer genes in systematic analyses of the breast cancer genome [31].
**UCP1**	uncoupling protein 1 (mitochondrial)	We conclude that UCP1 is up-regulated in breast cancer cell lines and primary breast as well as other tumors [32].
**PACSIN3**	Protein kinase C and casein kinase substrate in neurons protein 3	“New targets for breast cancer treatment were identified such as ZONAB, PACSIN3, MRP8 and SUMO1, which have human homologues” [33].
**PGM1**	phosphoglucomutase 1	“This gene has been identified as one of the ER status markers in the diagnosis and prognosis of breast cancer patients….” [34].
**TLN2**	talin 2	“Serum estradiol levels associated with specific gene expression patterns in normal breast tissue and in breast carcinomas……” [35].
**ADIPOR1**	adiponectin receptor 1	“Variants of the Adiponectin and Adiponectin Receptor 1 Genes and Breast Cancer Risk…” [36].
**PITX1**	paired-like homeodomain 1	“The estrogen-regulated transcription factor PITX1 coordinates gene-specific regulation by estrogen receptor-alpha in breast cancer cells…” [37].

**Table 6:** Genes from downregulated network

**Gene**	**Full name**	**Role in cancer quoted from literature**
**CELSR2**	cadherin, EGF LAG seven-pass G-type receptor 2	“Celsr2 was down-regulated in one cell line and in 7% of breast cancers” [38].
**PRKCB**	protein kinase C, beta	PRKCB has been observed to be downregulated in estrogen receptor negative breast cancer [39].
**KIT**	v-kit Hardy-Zuckerman 4 feline sarcoma viral oncogene homolog. Also known as C-Kit; CD117	Loss of c-kit expression has been reported in 80-90% of breast cancer specimens, suggesting a possible role in the development of tumors [40].
**MAP2**	microtubule-associated protein 2	“Elevation of MAP2 in breast cancer cell lines led to increased paclitaxel sensitivity” [41].
**TDGF1**	teratocarcinoma-derived growth factor 1. Also known as CR; CRGF; CRIPTO	“Overexpression of human Cripto-1 in transgenic mice delays mammary gland development and differentiation and induces mammary tumorigenesis” [42].
**ELF1**	E74-like factor 1	“All of the mouse and most human mammary tumors also displayed decreased expression of genes known to inhibit cell proliferation, including NFKBIA (IKBalpha), GADD45B, and CDKN1A (p21); transcription-related genes such as CEBP, JUN, JUNB, and ELF1;….” [43].
**RUNX1**	runt-related transcription factor 1	“Furthermore, we found that RUNX1 expression was reduced in high-grade primary breast tumors compared to low/mid-grade tumors” [44].
**TYMS**	thymidylate synthetase	“Gene polymorphisms in TYMS, MTHFR, p53 and MDR1 as risk factors for breast cancer: a case–control study” [45].

A number of genes identified with the downregulated networks are associated with breast cancer.

## Discussion

Mammary gland is sensitive to the carcinogenic actions of radiation and epidemiological data strongly correlates radiation exposure and breast cancer [1,2]. Here we demonstrate that exposure of 6 to 8 week old female mice to 2 Gy of γ radiation, a dose relevant to fractionated radiotherapy, induced long-term gene expression changes in the mammary glands. These changes are associated with molecular pathways that have the potential to enhance the chance of malignant transformation. We show that even after two months, a significant number of genes remain perturbed and that the majority of them are upregulated. Broadly, these genes are categorized into two major interdependent cellular functions – proliferation and metabolism. Upregulation of proliferative functions supported by induction of adaptive energy producing pathways is also observed in cancer cells [46]. Cancer, described as a chronic and multifactorial disease, was not observed in these mice within the study duration of 2 month. However, we did observe alterations in signaling pathways that may be considered potential carcinogenic precursors events.

The top network with a score of 44 associated 28 genes from the data set and literature indicates that at least 12 (ACHE, HSPA1L, MYOD1, NES, NOL3, P2RY2, PGK1, PLCG1, PPARGC1A, PRKCQ, S100B, and SNTA1) of them are involved in human cancer (genes relevant to breast cancer are presented with relevant references in Tables 5 and 6). Indeed, dysregulation of ACHE, HSPA1L, NES, P2RY2, PLCG1, PPARGC1A, PRKCQ, S100B, and SNTA1 genes have been implicated in breast cancer progression and metastasis due to higher proliferation and increase cell migration (Tables 5 and 6). Cellular metabolism and transportation related genes (ALDOA, SLC16A3, TPI1, LDHB, KCNQ5, and PGM1), which are implicated in human cancer, also remained upregulated even 2-month after radiation exposure. Importantly, induction of glycolytic enzymes like aldolase A (ALDOA), lactate dehydrogenase (LDH), triosephosphate isomerase 1 (TPI1), and phosphoglucomutase 1 (PGM1), we believe, is supporting cellular growth and development resulting from upregulated proliferative signaling pathways. It has been proposed that in a ‘Reverse Warburg Effect’ the glycolytic pathway is upregulated in stromal cells generating pyruvate/lactate, which can then be utilized by adjacent epithelial cells for oxidative phosphorylation in mitochondria [22]. Indeed, upregulation of ‘oxidative phosphorylation’ and ‘mitochondrial dysfunction’ are the two top canonical pathways observed in the IPA and are reported to be associated with breast cancer [47]. While stromal cell niche is known to play important roles in mammary gland carcinogenesis, we are yet to determine whether there are differential alterations of gene expression in stromal vs. epithelial cells and would require separate microarray analysis of stromal and epithelial compartment in mammary glands. However, considered along with the fact that in a whole boy irradiated mice all the tissue compartments are exposed to radiation, our results led us to speculate that differential alterations of gene expression in stromal vs. epithelial cells could occur in mammary gland to promote radiation carcinogenesis and is supportive of what has been reported in the literature [48,49]. Interestingly, both ALDOA and SLC16A3 are also suggested to be hypoxia responsive and is upregulated in hypoxic breast cancer with poor outcome. SLC16A3 is known to allow efflux of lactate [21], which we believe is produced in excess due to radiation-induced upregulation of glycolytic enzymes. Increased glycolysis may have served as a trigger for increased mitochondrial oxidative metabolism observed in our results, and could lead to oxidative stress as has been indicated by upregulation of canonical pathways involved in ‘mitochondrial dysfunction’ and ‘production of nitric oxide and reactive oxygen species in macrophages’. Interestingly, we also observed upregulation of UCP1 (uncoupling protein 1), a member of the UCP protein family known to uncouple respiratory chain events from ATP production. UPC1 is known to limit reactive oxygen species (ROS) production, which could result in oxidative stress below apoptotic threshold, and enhance cellular survival in the presence of irreversible injury to respiration [32,50]. The presence of radiation-induced mitochondrial dysfunction and consequent oxidative injury to cells are further exemplified by the upregulation of ENDOG, which has been shown to be involved in cell death in the absence of caspases [51].

Previously, we reported that exposure to 2 Gy radiation led to persistent increase in estrogen levels in serum and estrogen receptors in mammary glands at 2- and 12-month after radiation exposure and was associated with activation of PI3K-Akt proliferative pathways [50]. Microarray data showing upregulation of 17β hydroxysteroid dehydrogenase 7 (HSD17B7), an enzyme involved in conversion of weakly estrogenic estrone to biologically active estradiol in mammary glands, is reported to be induced by high serum estradiol [52] and is in agreement with our earlier observations of increased serum estradiol after radiation [50]. While systemic estradiol mediates its effects in mammary gland via ERα, higher expression of HSD17B7 could provide a ‘positive feed-forward’ mechanism for enhanced tissue estradiol level [28] and could explain activation of cellular proliferative signals observed by us [50]. Upregulation of estrogen-related receptor γ (ESRRG), which is induced by estrogen to enhance cell proliferation, provides further evidence of agreement between microarray data and our previous results. Importantly, even 2-month after exposure to 2 Gy radiation a high number of transcripts involved in cellular growth and development including strongly mitogenic MAPK12 remain significantly upregulated and are presented in Table 5.

Along with upregulation of genes involved in cellular growth and development, we also observed that a number of genes with tumor suppressor function (GPRC5A, ELF1, NAB2, Sema4D, ACPP, MAP2, RUNX1) persistently remained downregulated in response to radiation exposure further tilting the balance in favor of transformation (Table 6) [43,53-58]. In contrast, a few genes (TDGF1, GHRH, and BCL9) reported to be involved in promoting cell proliferation are downregulated which highlights the fact that cellular transformation is a multistage process with a sustained tug of war between tumor suppressor and tumor promoter. An important observation of our study was the downregulation of BRCC3 (BRCA1/BRCA2-containing complex, subunit 3), a protein known to play key a role in the repair of DNA damage through its E3 ubiquitin ligase activity on the repair factors [59] and may predispose the mammary gland cells to misrepair and genomic instability. A number of other downregulated genes positively affecting cellular growth and proliferation have been observed in our study and are listed in Table 6.

Molecular pathway networks identified by IPA allowed us to understand how genes in our data sets spatially relate to oncogenic precursor events relevant to breast cancer. The top-scoring pathway has NFkβ and Akt (Figure 4A) as the central molecules and both are known to be involved in breast carcinogenesis [60,61]. Another high scoring upregulated network identified ERBB2, an important membrane bound receptor tyrosine kinase, as the nodal molecule (Figure 8B) and is known to crosstalk with NFkβ and PI3K-Akt to promote breast cancer [62]. One of the important observations in our study was the recognition of inflammation related factors in the IPA network and a number of pathway showed IL-5 (Figure 4B), IL-1β (Figure 6B), TNF (Figure 7A), IL-4, and INFγ (Figure 7B) as the central molecules highlighting an already existing relationship between inflammation and breast cancer [63,64]. Reproductive hormones LH and FSH are the central molecules in an upregulated pathway network (Figure 5A) and are known to regulate estrogen [65], which is strongly associated with risk of developing breast cancer [66]. Indeed, in an earlier study, we have shown that exposure to a radiation dose (2 Gy) used in the current study significantly increased serum estrogen level, urinary oncogenic estrogen metabolite level, mammary gland estrogen receptor α (ERα) level, and cellular proliferation in mice [50]. Estrogen has also been reported to influence TGFβ activity negatively [67], and interestingly our downregulated data sets mapped to a pathway, which has TGFβ as the nodal molecule (Figure 10A). In our earlier study, we focused on exploring one pathway, the PI3K-Akt pathway, in relation to increased estrogen and ERα and showed that radiation not only upregulated PI3K and Akt activity but also induced their downstream effectors leading to increased mammary gland cell proliferation determined by phospho-histone H3 staining [50]. Current microarray based complete gene expression study after radiation exposure not only showed upregulation of additional pathways already discussed but also showed increased expression of genes associated with pathways having TP53 (Figure 6A), AGTR1 (Figure 8A), P38-MAPK and PLCG1 (Figure 9B), PI3K complex and MAPK3 (Figure 9A) as central molecule. The molecular pathways, which IPA identified to be significantly associated with the genes from the microarray data sets have been implicated in human cancer including breast cancer (Tables 5 and 6) and provide evidence towards carcinogenic potential of therapeutic radiation exposure.

## Conclusions

Exposure to diagnostic and therapeutic radiation has increased considerably over time [3,68,69] and knowledge of the molecular pathways that remain perturbed long-term after radiation exposure could inform us to devise strategies to tackle the carcinogenic potential of radiation. Taken together the microarray data presented here not only correlate with our earlier observations on long-term effects of radiation exposure on mammary gland pathophysiology, but they also further expand on the persistently perturbed molecular networks with potential for transformation after exposure to a clinically relevant radiation dose.

## Competing interests

Authors have no conflict of interest to declare.

## Authors’ contributions

KD: planned and executed experiments, analyzed results, and prepared the manuscript; DH: executed experiments and analyzed results; SS: executed experiments and analyzed results; MJ: participated in preparing the manuscript; AF: participated in preparing the manuscript. All authors read and approved the final manuscript.

## Supplementary Material

Additional file 1 **All the upregulated canonical pathways mapped by Ingenuity Pathway Analysis are presented.** The colored (blue) pathways have p-value <0.05. Click here for file

Additional file 2 **All the downregulated canonical pathways mapped by Ingenuity Pathway Analysis are presented.** The colored (blue) pathways have p-value <0.05. Click here for file

Additional file 3 **Complete list of upregulated signaling networks generated by Ingenuity Pathway Analysis are presented.** Highlighted (bold) genes are from the microarray dataset. Click here for file

Additional file 4 **Complete list of downregulated signaling networks generated by Ingenuity Pathway Analysis are presented.** Highlighted (bold) genes are from the microarray dataset. Click here for file

## References

[B1] Ronckers CM Erdmann CA Land CE Radiation and breast cancer: a review of current evidence Breast Cancer Res 2005 7 21 32 10.1186/bcr970 15642178 PMC1064116

[B2] Shuryak I Sachs RK Brenner DJ Cancer risks after radiation exposure in middle age J Natl Cancer Inst 2010 102 1628 1636 10.1093/jnci/djq346 20975037 PMC2970575

[B3] Brenner DJ Hricak H Radiation exposure from medical imaging: time to regulate JAMA 2010 304 208 209 10.1001/jama.2010.973 20628137

[B4] Boice JDJ Harvey EB Blettner M Stovall M Flannery JT Cancer in the contralateral breast after radiotherapy for breast cancer N Engl J Med 1992 326 781 785 10.1056/NEJM199203193261201 1538720

[B5] Snyder AR Morgan WF Gene expression profiling after irradiation: clues to understanding acute and persistent responses Cancer Metastasis Rev 2004 23 259 268 15197327 10.1023/B:CANC.0000031765.17886.fa

[B6] Prolla TA DNA microarray analysis of the aging brain Chem Senses 2002 27 299 306 10.1093/chemse/27.3.299 11923192

[B7] Benjamini Y Hochberg Y Controlling the false discovery rate: A practical and powerful approach to multiple testing Journal of the Royal Statistical Society, Series B (Methodological) 1995 57 289 300

[B8] Gupta RK Arany Z Seale P Mepani RJ Ye L Conroe HM Roby YA Kulaga H Reed RR Spiegelman BM Transcriptional control of preadipocyte determination by Zfp423 Nature 2010 464 619 623 10.1038/nature08816 20200519 PMC2845731

[B9] Gubelmann C Gattiker A Massouras A Hens K David F Decouttere F Rougemont J Deplancke B GETPrime: a gene- or transcript-specific primer database for quantitative real-time PCR Database (Oxford) 2011 2011 bar040 21917859 10.1093/database/bar040 PMC3173022

[B10] Livak KJ Schmittgen TD Analysis of relative gene expression data using real-time quantitative PCR and the 2(-Delta Delta C(T)) Method Methods 2001 25 402 408 10.1006/meth.2001.1262 11846609

[B11] Bernardi C Ribeiro C Ede S Cavalli IJ Chautard-Freire-Maia EA Souza RL Amplification and deletion of the ACHE and BCHE cholinesterase genes in sporadic breast cancer Cancer Genet Cytogenet 2010 197 158 165 10.1016/j.cancergencyto.2009.10.011 20193849

[B12] Zajac M Gomez G Benitez J Martinez-Delgado B Molecular signature of response and potential pathways related to resistance to the HSP90 inhibitor, 17AAG, in breast cancer BMC Med Genomics 2010 3 44 10.1186/1755-8794-3-44 20920318 PMC2959047

[B13] Ishiwata T Matsuda Y Naito Z Nestin in gastrointestinal and other cancers: effects on cells and tumor angiogenesis World J Gastroenterol 2011 17 409 418 10.3748/wjg.v17.i4.409 21274370 PMC3027007

[B14] Li HJ Wang LY Qu HN Yu LH Burnstock G Ni X Xu M Ma B P2Y2 receptor-mediated modulation of estrogen-induced proliferation of breast cancer cells Mol Cell Endocrinol 2011 338 28 37 10.1016/j.mce.2011.02.014 21356271

[B15] Sala G Dituri F Raimondi C Previdi S Maffucci T Mazzoletti M Rossi C Iezzi M Lattanzio R Piantelli M Phospholipase Cgamma1 is required for metastasis development and progression Cancer Res 2008 68 10187 10196 10.1158/0008-5472.CAN-08-1181 19074886

[B16] Wirtenberger M Tchatchou S Hemminki K Schmutzhard J Sutter C Schmutzler RK Meindl A Wappenschmidt B Kiechle M Arnold N Associations of genetic variants in the estrogen receptor coactivators PPARGC1A, PPARGC1B and EP300 with familial breast cancer Carcinogenesis 2006 27 2201 2208 10.1093/carcin/bgl067 16704985

[B17] Belguise K Sonenshein GE PKCtheta promotes c-Rel-driven mammary tumorigenesis in mice and humans by repressing estrogen receptor alpha synthesis J Clin Invest 2007 117 4009 4021 18037997 10.1172/JCI32424 PMC2082145

[B18] Bhat HF Baba RA Bashir M Saeed S Kirmani D Wani MM Wani NA Wani KA Khanday FA Alpha-1-syntrophin protein is differentially expressed in human cancers Biomarkers 2011 16 31 36 10.3109/1354750X.2010.522731 21091386

[B19] Cortesi L Barchetti A De Matteis E Rossi E Della Casa L Marcheselli L Tazzioli G Lazzaretti MG Ficarra G Federico M Iannone A Identification of protein clusters predictive of response to chemotherapy in breast cancer patients J Proteome Res 2009 8 4916 4933 10.1021/pr900239h 19739612

[B20] Garcia-Marcos M Ghosh P Farquhar MG Molecular basis of a novel oncogenic mutation in GNAO1 Oncogene 2011 30 2691 2696 10.1038/onc.2010.645 21317923 PMC3349449

[B21] Hu Z Fan C Livasy C He X Oh DS Ewend MG Carey LA Subramanian S West R Lkpatt F A compact VEGF signature associated with distant metastases and poor outcomes BMC Med 2009 7 9 10.1186/1741-7015-7-9 19291283 PMC2671523

[B22] Migneco G Whitaker-Menezes D Chiavarina B Castello-Cros R Pavlides S Pestell RG Fatatis A Flomenberg N Tsirigos A Howell A Glycolytic cancer associated fibroblasts promote breast cancer tumor growth, without a measurable increase in angiogenesis: evidence for stromal-epithelial metabolic coupling Cell Cycle 2010 9 2412 2422 10.4161/cc.9.12.11989 20562527

[B23] Dorssers LC van Agthoven T Brinkman A Veldscholte J Smid M Dechering KJ Breast cancer oestrogen independence mediated by BCAR1 or BCAR3 genes is transmitted through mechanisms distinct from the oestrogen receptor signalling pathway or the epidermal growth factor receptor signalling pathway Breast Cancer Res 2005 7 R82 92 10.1186/bcr954 15642172 PMC1064102

[B24] Reimer CL Borras AM Kurdistani SK Garreau JR Chung M Aaronson SA Lee SW Altered regulation of cyclin G in human breast cancer and its specific localization at replication foci in response to DNA damage in p53+/+ cells J Biol Chem 1999 274 11022 11029 10.1074/jbc.274.16.11022 10196184

[B25] Oberley MJ Inman DR Farnham PJ E2F6 negatively regulates BRCA1 in human cancer cells without methylation of histone H3 on lysine 9 J Biol Chem 2003 278 42466 42476 10.1074/jbc.M307733200 12909625

[B26] Ijichi N Shigekawa T Ikeda K Horie-Inoue K Fujimura T Tsuda H Osaki A Saeki T Inoue S Estrogen-related receptor gamma modulates cell proliferation and estrogen signaling in breast cancer J Steroid Biochem Mol Biol 2011 123 1 7 10.1016/j.jsbmb.2010.09.002 20883782

[B27] Rosenthal DT Iyer H Escudero S Bao L Wu Z Ventura AC Kleer CG Arruda EM Garikipati K Merajver SD p38gamma promotes breast cancer cell motility and metastasis through regulation of RhoC GTPase, cytoskeletal architecture, and a novel leading edge behavior Cancer Res 2011 71 6338 6349 10.1158/0008-5472.CAN-11-1291 21862636 PMC3193559

[B28] Shehu A Albarracin C Devi YS Luther K Halperin J Le J Mao J Duan RW Frasor J Gibori G The stimulation of HSD17B7 expression by estradiol provides a powerful feed-forward mechanism for estradiol biosynthesis in breast cancer cells Mol Endocrinol 2011 25 754 766 10.1210/me.2010-0261 21372145 PMC3082328

[B29] Cheng S Douglas-Jones A Yang X Mansel RE Jiang WG Transforming acidic coiled-coil-containing protein 2 (TACC2) in human breast cancer, expression pattern and clinical/prognostic relevance Cancer Genomics Proteomics 2010 7 67 73 20335520

[B30] Germain DR Graham K Glubrecht DD Hugh JC Mackey JR Godbout R DEAD box 1: a novel and independent prognostic marker for early recurrence in breast cancer Breast Cancer Res Treat 2011 127 53 63 10.1007/s10549-010-0943-7 20499159

[B31] Alhopuro P Karhu A Winqvist R Waltering K Visakorpi T Aaltonen LA Somatic mutation analysis of MYH11 in breast and prostate cancer BMC Cancer 2008 8 263 10.1186/1471-2407-8-263 18796164 PMC2562392

[B32] Ayyasamy V Owens KM Desouki MM Liang P Bakin A Thangaraj K Buchsbaum DJ LoBuglio AF Singh KK Cellular model of Warburg effect identifies tumor promoting function of UCP2 in breast cancer and its suppression by genipin PLoS One 2011 6 e24792 10.1371/journal.pone.0024792 21935467 PMC3174207

[B33] Sharp JA Mailer SL Thomson PC Lefevre C Nicholas KR Sharp JA Mailer SL Thomson PC Lefevre C Nicholas KR Identification and transcript analysis of a novel wallaby (Macropus eugenii) basal-like breast cancer cell line Mol Cancer 2008 7 1 18179684 10.1186/1476-4598-7-1 PMC2263075

[B34] Ma S Kosorok MR Detection of gene pathways with predictive power for breast cancer prognosis BMC Bioinformatics 2010 11 1 10.1186/1471-2105-11-1 20043860 PMC2837025

[B35] Haakensen VD Bjoro T Luders T Riis M Bukholm IK Kristensen VN Troester MA Homen MM Ursin G Borresen-Dale AL Helland A Serum estradiol levels associated with specific gene expression patterns in normal breast tissue and in breast carcinomas BMC Cancer 2011 11 332 10.1186/1471-2407-11-332 21812955 PMC3163631

[B36] Kaklamani VG Sadim M Hsi A Offit K Oddoux C Ostrer H Ahsan H Pasche B Mantzoros C Variants of the adiponectin and adiponectin receptor 1 genes and breast cancer risk Cancer Res 2008 68 3178 3184 10.1158/0008-5472.CAN-08-0533 18451143 PMC2685173

[B37] Stender JD Stossi F Funk CC Charn TH Barnett DH Katzenellenbogen BS The estrogen-regulated transcription factor PITX1 coordinates gene-specific regulation by estrogen receptor-alpha in breast cancer cells Mol Endocrinol 2011 25 1699 1709 10.1210/me.2011-0102 21868451 PMC3182422

[B38] Huang H Groth J Sossey-Alaoui K Hawthorn L Beall S Geradts J Aberrant expression of novel and previously described cell membrane markers in human breast cancer cell lines and tumors Clin Cancer Res 2005 11 4357 4364 10.1158/1078-0432.CCR-04-2107 15958618

[B39] Naderi A Meyer M Dowhan DH Cross-regulation between FOXA1 and ErbB2 signaling in estrogen receptor-negative breast cancer Neoplasia 2012 14 283 296 22577344 10.1593/neo.12294 PMC3349255

[B40] Nishida K Tsukamoto T Uchida K Takahashi T Takahashi T Ueda R Introduction of the c-kit gene leads to growth suppression of a breast cancer cell line, MCF-7 Anticancer Res 1996 16 3397 3402 9042197

[B41] Bauer JA Chakravarthy AB Rosenbluth JM Mi D Seeley EH De Matos Granja-Ingram N Olivares MG Kelley MC Mayer IA Meszoely IM Identification of markers of taxane sensitivity using proteomic and genomic analyses of breast tumors from patients receiving neoadjuvant paclitaxel and radiation Clin Cancer Res 2010 16 681 690 10.1158/1078-0432.CCR-09-1091 20068102 PMC2892225

[B42] Sun Y Strizzi L Raafat A Hirota M Bianco C Feigenbaum L Kenney N Wechselberger C Callahan R Salomon DS Overexpression of human Cripto-1 in transgenic mice delays mammary gland development and differentiation and induces mammary tumorigenesis Am J Pathol 2005 167 585 597 10.1016/S0002-9440(10)63000-3 16049342 PMC1603555

[B43] Hu Y Sun H Drake J Kittrell F Abba MC Deng L Gaddis S Sahin A Baggerly K Medina D Aldaz CM From mice to humans: identification of commonly deregulated genes in mammary cancer via comparative SAGE studies Cancer Res 2004 64 7748 7755 10.1158/0008-5472.CAN-04-1827 15520179 PMC4170686

[B44] Kadota M Yang HH Gomez B Sato M Clifford RJ Meerzaman D Dunn BK Wakefield LM Lee MP Delineating genetic alterations for tumor progression in the MCF10A series of breast cancer cell lines PLoS One 2010 5 e9201 10.1371/journal.pone.0009201 20169162 PMC2821407

[B45] Henriquez-Hernandez LA Murias-Rosales A Hernandez Gonzalez A Cabrera De Leon A Diaz-Chico BN Mori De Santiago M Fernandez Perez L Gene polymorphisms in TYMS, MTHFR, p53 and MDR1 as risk factors for breast cancer: a case-control study Oncol Rep 2009 22 1425 1433 19885596 10.3892/or_00000584

[B46] DeBerardinis RJ Lum JJ Hatzivassiliou G Thompson CB The biology of cancer: metabolic reprogramming fuels cell growth and proliferation Cell Metab 2008 7 11 20 10.1016/j.cmet.2007.10.002 18177721

[B47] Whitaker-Menezes D Martinez-Outschoorn UE Flomenberg N Birbe RC Witkiewicz AK Howell A Pavlides S Tsirigos A Ertel A Pestell RG Hyperactivation of oxidative mitochondrial metabolism in epithelial cancer cells in situ: Visualizing the therapeutic effects of metformin in tumor tissue Cell Cycle 2011 10 4047 4064 10.4161/cc.10.23.18151 22134189 PMC3272287

[B48] Allinen M Beroukhim R Cai L Brennan C Lahti-Domenici J Huang H Porter D Hu M Chin L Richardson A Molecular characterization of the tumor microenvironment in breast cancer Cancer Cell 2004 6 17 32 10.1016/j.ccr.2004.06.010 15261139

[B49] Huang M Li Y Zhang H Nan F Breast cancer stromal fibroblasts promote the generation of CD44+CD24- cells through SDF-1/CXCR4 interaction J Exp Clin Cancer Res 2010 29 80 10.1186/1756-9966-29-80 20569497 PMC2911413

[B50] Suman S Johnson MD Fornace AJJ Datta K Exposure to Ionizing Radiation Causes Long-Term Increase in Serum Estradiol and Activation of PI3K-Akt Signaling Pathway in Mouse Mammary Gland Int J Radiat Oncol Biol Phys 2012 84 500 507 10.1016/j.ijrobp.2011.12.033 22381906 PMC3580184

[B51] Higgins GC Beart PM Nagley P Oxidative stress triggers neuronal caspase-independent death: endonuclease G involvement in programmed cell death-type III Cell Mol Life Sci 2009 66 2773 2787 10.1007/s00018-009-0079-2 19582370 PMC11115579

[B52] Haynes BP Straume AH Geisler J A'Hern R Helle H Smith IE Lonning PE Dowsett M Intratumoral estrogen disposition in breast cancer Clin Cancer Res 2010 16 1790 1801 10.1158/1078-0432.CCR-09-2481 20215536

[B53] Abdulkadir SA Carbone JM Naughton CK Humphrey PA Catalona WJ Milbrandt J Frequent and early loss of the EGR1 corepressor NAB2 in human prostate carcinoma Hum Pathol 2001 32 935 939 10.1053/hupa.2001.27102 11567222

[B54] Kadara H Fujimoto J Men T Ye X Lotan D Lee JS Lotan R A Gprc5a tumor suppressor loss of expression signature is conserved, prevalent, and associated with survival in human lung adenocarcinomas Neoplasia 2010 12 499 505 20563252 10.1593/neo.10390 PMC2887090

[B55] Soong J Chen Y Shustef EM Scott GA Sema4D, the Ligand for Plexin B1, Suppresses c-Met Activation and Migration and Promotes Melanocyte Survival and Growth J Invest Dermatol 2012 132 1230 1238 10.1038/jid.2011.414 22189792 PMC3305852

[B56] Veeramani S Yuan TC Chen SJ Lin FF Petersen JE Shaheduzzaman S Srivastava S MacDonald RG Lin MF Cellular prostatic acid phosphatase: a protein tyrosine phosphatase involved in androgen-independent proliferation of prostate cancer Endocr Relat Cancer 2005 12 805 822 10.1677/erc.1.00950 16322323

[B57] Song Z He CD Sun C Xu Y Jin X Zhang Y Xiao T Wang Y Lu P Jiang Y Increased expression of MAP2 inhibits melanoma cell proliferation, invasion and tumor growth in vitro and in vivo Exp Dermatol 2010 19 958 964 10.1111/j.1600-0625.2009.01020.x 20100193

[B58] Silva FP Morolli B Storlazzi CT Anelli L Wessels H Bezrookove V Kluin-Nelemans HC Giphart-Gassler M Identification of RUNX1/AML1 as a classical tumor suppressor gene Oncogene 2003 22 538 547 10.1038/sj.onc.1206141 12555067

[B59] Dong Y Hakimi MA Chen X Kumaraswamy E Cooch NS Godwin AK Shiekhattar R Regulation of BRCC, a holoenzyme complex containing BRCA1 and BRCA2, by a signalosome-like subunit and its role in DNA repair Mol Cell 2003 12 1087 1099 10.1016/S1097-2765(03)00424-6 14636569

[B60] Shostak K Chariot A NF-kappaB, stem cells and breast cancer: the links get stronger Breast Cancer Res 2011 13 214 10.1186/bcr2886 21867572 PMC3236328

[B61] Liu W Bagaitkar J Watabe K Roles of AKT signal in breast cancer Front Biosci 2007 12 4011 4019 10.2741/2367 17485354

[B62] Zhou Y Eppenberger-Castori S Eppenberger U Benz CC The NFkappaB pathway and endocrine-resistant breast cancer Endocr Relat Cancer 2005 1 12 S37 46 16113098 10.1677/erc.1.00977

[B63] Coussens LM Werb Z Inflammation and cancer Nature 2002 420 860 867 10.1038/nature01322 12490959 PMC2803035

[B64] Mantovani A Marchesi F Porta C Sica A Allavena P Inflammation and cancer: breast cancer as a prototype Breast 2007 2 16 S27 33 17764938 10.1016/j.breast.2007.07.013

[B65] Powell BL Piersma D Kevenaar ME van Staveren IL Themmen AP Iacopetta BJ Berns EM Luteinizing hormone signaling and breast cancer: polymorphisms and age of onset J Clin Endocrinol Metab 2003 88 1653 1657 10.1210/jc.2002-021585 12679452

[B66] Pike MC Spicer DV Dahmoush L Press MF Estrogens, progestogens, normal breast cell proliferation, and breast cancer risk Epidemiol Rev 1993 15 17 35 8405201 10.1093/oxfordjournals.epirev.a036102

[B67] Hinshelwood RA Huschtscha LI Melki J Stirzaker C Abdipranoto A Vissel B Ravasi T Wells CA Hume DA Reddel RR Clark SJ Concordant epigenetic silencing of transforming growth factor-beta signaling pathway genes occurs early in breast carcinogenesis Cancer Res 2007 67 11517 11527 10.1158/0008-5472.CAN-07-1284 18089780

[B68] Kleinerman RA Cancer risks following diagnostic and therapeutic radiation exposure in children Pediatr Radiol 2006 36 Suppl 2 121 125 16862418 10.1007/s00247-006-0191-5 PMC2663653

[B69] Mettler FAJ Voelz GL Major radiation exposure--what to expect and how to respond N Engl J Med 2002 346 1554 1561 10.1056/NEJMra000365 12015396

